# Risk Factors of Postpartum Depression

**DOI:** 10.7759/cureus.30898

**Published:** 2022-10-31

**Authors:** Iris Agrawal, Ashok M Mehendale, Ritika Malhotra

**Affiliations:** 1 Department of Community Medicine, Jawaharlal Nehru Medical College, Datta Meghe Institute of Medical Sciences, Wardha, IND

**Keywords:** postnatal depression, postpartum care, postpartum mothers, postpartum mental health, post-natal mother, history of depression, spousal support, clinical features, risk factors, postpartum depression

## Abstract

Postpartum depression (PPD) is a widespread mental health problem and one of the prime causes of maternal suffering and ill health. On a global level, the prevalence of the disorder is about 10 to 15%. Symptoms generally appear within the first four to six weeks, which is the high-risk period. However, it may develop up to one year post-delivery. PPD presents with symptoms of classical depression, including mood fluctuations, bouts of crying, lack of interest in the child, and even thoughts of suicide. PPD not only has adverse effects on the mother’s health but also hampers the growth and development of the child. It hampers the formation of a healthy mother-child bond, which in turn may impact feeding practices. The social environment of the infant during the first few months is primarily provided by the mother, and PPD may thus impact the child’s development. It also increases the child’s susceptibility to malnutrition.

Research on postpartum depression has garnered momentum within the last few years. However, the masses are still largely unaware of the disorder and its implications. There is also an inadequacy of awareness of the risk factors of PPD. The cross-cultural differences in manifestations and appropriate preventive measures have not been extensively studied. Some risk factors for PPD are similar to those for classic depression; however, obstetrical and pediatric factors are also involved.

This literature review aims to assess the currently known risk factors for PPD, their strength of association, and probable mechanisms to help identify the high-risk group and enable the implementation of preventive measures or facilitate early diagnosis. The factors identified spanned sociodemographic, biological, psychological, and obstetric domains. These included socioeconomic standing, marital relationship, history of psychiatric illness, social support, gestational diabetes, vitamin D deficiency, immigration status, delivery method, violence and abuse, birth experience, and biological and epigenetic markers. The risk factors for postpartum depression are numerous and may have strong to weak associations with the development of PPD. A previous history of depression or psychiatric illness, depressive symptoms during pregnancy, gestational diabetes, and a lack of spousal and social support were the most powerful risk factors. Other significant factors include complications during pregnancy, low socioeconomic status, and stressful life events. Studies on maternal age and chronic illness as risk factors were inconclusive. The roles of genetic and epigenetic markers, cultural factors, and vitamin D insufficiency require further investigation.

## Introduction and background

Pregnancy and motherhood are the transformative periods of a woman’s life, signifying an enormous change from a psychological, physical, and social standpoint, with women encountering many difficulties, such as learning several parenting techniques, forging new familial bonds, and undergoing significant physical and psychological adjustments [1-3]. Psychological vulnerability is especially seen during postpartum. Consequently, there is a high prevalence of bonding and mood disorders within this period, the most common being postpartum depression, whose incidence, according to a recent study, came out to be 24% among healthy women in the post-delivery period [4,5]. A major depressive episode during the postpartum period is referred to as postpartum depression (PPD) [6]. A moderate to severe depressive episode commencing four weeks post-delivery is referred to as postpartum depression according to the Diagnostic and Statistical Manual of Mental Disorders, Fifth Edition (DSM-5). PPD is defined as a disorder and a form of depression by the World Health Organization (WHO) [7]. The primary symptoms appear four to eight weeks post-delivery [1].

It can be confusing and upsetting to experience the conflict between the positive sentiments that mothers are frequently made to believe and even expected to feel and the depressive mood and worry that several of them experience. Many women believe such symptoms to be a passing phenomenon, not requiring medical intervention, which is true for a case of postpartum blues, occurring within the first ten days of delivery and characterized by mild mood disturbance [8,9]. However, postpartum depression (PPD) is a clinical disorder that significantly impairs functioning, frequently necessitates professional care, and typically lasts two weeks [10].

According to current statistics, about 85% of women face emotional disturbances during postpartum [11]. Subjective to the diagnostic criteria, the frequency of occurrence of PPD varies from 10% to 15% to as high as 30% [12-14]. Prior studies have indicated the prevalence of PPD worldwide till 2017 to range from roughly 9.5% in high-income nations to 20.8% in middle-income regions to around 25.8% in low-income nations [15,16]. In India, postpartum depression is seen to be a frequent occurrence. According to recent statistics, its national incidence is between 19.8% and 23.3% [17]. The majority of PPD sufferers may recoup in a few months. However, about 30% of them may continue to struggle with depression even after a year of giving birth. Additionally, the probability of recurrence following postpartum or non-postpartum patients was about 40% [18]. The following literature review aims to highlight the numerous risk factors that may predispose women to postpartum depression.

## Review

Search methodology

A search was initiated in the PubMed database using the keywords "postpartum depression", "risk factors", and "predisposing factors". The number of articles derived from the search was 5,383. After applying the relevant inclusion and exclusion criteria, a total of 77 articles were shortlisted and exported to Zotero. Articles including original articles, case-control studies, systematic reviews, meta-analyses, and retrospective and longitudinal studies published in English that had researched risk factors for postpartum depression were included in the review. After careful screening of articles, a total of 58 articles were included in the final analysis.

Clinical presentation

Postpartum depression (PPD) comprises the classic signs of a depressive episode as well as unreasonable worries about the child's health, suicidal thoughts, and occasional thoughts of infanticide. Typically starting between the second and sixth week after birth, depressive symptoms can emerge at any point throughout the first to twelfth month of postpartum. The severity and prognosis of these symptoms are strongly related to the time of diagnosis and therapy [19]. Bouts of crying, anxiety, mood fluctuations, sleep disturbances, appetite loss, thoughts of injuring oneself or one’s baby, as well as a lack of interest in the infant are some of the warning symptoms of PPD in women [17].

The effect of PPD on mothers and children

It is astonishing that despite the disease's enormous prevalence and seriousness around the world, around 80% of cases go undiagnosed and untreated, with catastrophic long-term effects on the entire family, especially the mother and her child. Notable risks include an increased likelihood of severe and persistent depression in the mother, challenges with parenting, and suicide risk [19]. PPD has been found to have a profound negative impact on mothers as well as their children. The mother-child relationship is also adversely impacted [17]. Children of depressed mothers are more prone to being underweight and stunted and face a higher incidence of physical issues, according to studies that have highlighted the ramifications of PPD on the development of children. Another comprehensive qualitative study demonstrated that mothers suffering from PPD might be more prone to poor infant feeding practices, negatively affecting the mother-to-child bond [20]. It is linked to a severe lack of well-being, and the child's cognitive, emotional, intellectual, and social development may be impacted in the long run [21].

Risk factors

The identification of agents posing and elevating the risk of developing PPD in mothers has been the subject of extensive research. A literature review and meta-analysis carried out by Zhao et al. found 48 articles identifying factors having a positive correlation with the development of postpartum depression. These include delivery by cesarean section, gestational diabetes, violence and abuse, socioeconomic status, immigration, lack of spousal and societal support, past depressive history, factors causing depression, unwanted pregnancy, obese and overweight mothers, vitamin D deficiency, diet and nutrition, parity, infant-related factors, sleep-related factors in the postpartum period, and postpartum anemia [20]. Other factors are age, marital status, maternal health before pregnancy, stressful life events, childcare stress, lack of breastfeeding, previous abortions, negative pregnancy and/or delivery experience, baby's gender, smoking, and alcohol or substance abuse [19,22,23].

Numerous factors have thus been implicated as being positively correlated with PPD across various studies. As described by Klainin et al., the risk factors in this review have been classified into sociodemographic, physical and biological, psychological, obstetric and pediatric, and cultural factors [24]. The risk factors and their relationships with PPD as found by different studies are given in Table 1.

**Table 1:** A summary of systematic reviews of risk factors for postpartum depression. [20]

Risk factor	Findings	Study designs
Social relationships	Low spousal and social support were implicated as risk factors for PPD.	Five longitudinal studies, three cross-sectional studies, and one qualitative in-depth interview were conducted.
Past history of depression and lack of support	Most studies show an elevated odds ratio between lack of support and past depression and the occurrence of PPD.	Two RCTs; one population-based survey; one telephonic survey; one minority patient chart review; one prospective longitudinal study; one systematic review and one meta-analysis.
Immigration status	Higher levels of acculturation were reported by five studies as a risk factor for PPD.	Four cross-sectional studies and three longitudinal studies
Genetic and epigenetic markers	The studies analyzed and outlined associations between postpartum depression and epigenetic marker modifications, SNPs, and deletion or insertion polymorphisms.	37 studies
Chronic illness	Not much evidence was found.	Four studies
Vitamin D	Out of the nine studies, five showed a positive correlation between vitamin D in pregnancy and PPD, while the others showed no correlation.	Six prospective studies; four cross-sectional studies; two prospective secondary analyses; one RCT; one case-control study
Cesarean section	PPD levels did not differ significantly between women who had an emergency cesarean section and those who had a normal vaginal delivery.	Three cohorts
Abuse	PPD was higher among those with past or current abuse as well as those with substance abuse disorders.	Eight designs
Diabetes	In both PPD and depression in pregnancy, diabetes was a risk factor.	15 observational retrospective designs were described, 30 prospective observational designs were described, and three RCTs were described.
Body image dissatisfaction	A consistent but weak association was found.	Nine prospective cohorts, 10 cross-sectional studies
Multiple births	Higher levels of PPD were seen.	Seven studies
Women’s birth experiences	11 studies demonstrated an increase in the occurrence of PPD in women with negative birth experiences.	Five studies were retrospective; five were secondary analyses of previously collected data; and five were prospective.
Pre-pregnancy obesity	Two studies reported a positive correlation, while one reported no association.	Pre-pregnancy obesity
Women with preterm and low-birth-weight infants	About 40% of women with preterm deliveries develop PPD early in the postpartum period. Women with LBW babies have a higher risk of developing PPD till a year after delivery.	26 studies

Sociodemographic factors

Evidence of the connection between the age of the mother and PPD varies. Some young mothers aged less than 25 years were found to be at greater risk for PPD by some studies [25-29]. One study reported a threefold risk in postpartum women under 25 years of age of developing PPD [30]. However, numerous other studies concluded that there was no correlation between the two [31-34].

The relationship status of the mother, including formal marital status, "de facto" marriage, or the presence of any relationship with the baby's biological father, is one of the most frequently studied factors in PPD. However, according to two meta-analyses, marital status is not as significant a factor in PPD as is commonly believed [35,36]. One study observed that while marital status did appear to be a risk factor at the outset, once the relationship status was controlled, it became inconsequential [37].

Lower socioeconomic status is indicated by factors like low income, lower level of education, low literacy, and unemployment, which have been implicated as risk factors for PPD, especially in developing countries [21]. It has been suggested that a lack of education might be linked to a lack of information and understanding of effective methods for postpartum care and childrearing. At the same time, financial limitations may result in decreased expenditures on the mother's health and well-being [34,38,39].

Immigration is a complicated risk factor for PPD; it does not act as a standalone but rather is influenced by other factors, including social support, acculturation, and economic standing, which play a significant part. More particularly, acculturation, limited social support, poor socioeconomic position, and other issues associated with immigration influence PPD, not the immigrant status in and of itself [20].

Physical and biological factors

According to recent studies investigating the topic, there may be a connection between PPD and being overweight or obese, which has become a global health issue. The level of obesity was positively correlated with the risk of PPD. Another factor closely associated with obesity, body image, has also been studied, and evidence suggests a positive association, albeit weak, between dissatisfaction with body image and the incidence of PPD [20,40]. According to a separate study, women who are obese may experience higher levels of inflammation and stress. These are two factors implicated in depression [41].

The risk of PPD is considered to increase in mothers with poor physical health or those having a history of severe medical illness [28,42,43]. However, literature on their association is significantly lacking.

In the face of stressful life events, polymorphisms in the HMNC1, COMT, MAOT, PRKCB, ESR1, and SLC6A4 genes, in the brain-derived neurotrophic factor (BDNF) gene when the postpartum phase occurs in the months of fall and winter, and the OXT and OXTR genes when mothers who had experienced difficulties as children reportedly had a significant connection with postpartum depression. The conclusion was drawn by Elwood et al. in a review investigating the role of genetic and epigenetic markers in PPD [43,44].

Psychological factors

Suffering from depression or anxiety during pregnancy or having a history of depressive disorders is among the strongest predictors of postpartum depression. Prediction of the onset and progression of various symptoms of PPD was found to be strongly associated with higher anxiety levels. The evidence to date from studies conducted on a large scale points to a positive relationship between having a family history of psychiatric illness and PPD [36,44]. A meta-analysis conducted in 2001 analyzed the strength of the association of risk of PPD with prenatal depression and prior history of depression and found both to be in the medium effect range, with r being 0.48 and 0.38, respectively [45]. It has repeatedly been observed that past depression is a significant and among the most potent risk factors for PPD. PPD has a 1:3 probability of developing in women who have a history of suffering from some mood disorder. It has also been noticed that women who had suffered from depression in the past had a 30% higher risk of PPD, which is considerably higher compared to women with no such history of psychiatric illness. It's noteworthy that such women also had a far higher prevalence of postpartum blues than those who had no history of affective disorders [46,47]. While antenatal depression is a confirmed risk factor, investigations regarding the basis of this association, whether it is psychosocial or a consequence of genetic vulnerability, have yet to provide concrete evidence [48].

A recently observed but fairly significant predictor of PPD, self-esteem, was found to lower the negative emotions attached to stressful life events. Depression, on the other hand, is believed to be triggered by low self-esteem [30]. Mothers with higher self-esteem are better equipped to handle pressures that could compromise their image of themselves and self-esteem and lead to postpartum depression. However, Sichel et al. warned that during postpartum, a highly vulnerable time, the self-esteem of even the strongest women could worsen due to depression [45].

It has been well established that stressful life circumstances might cause the development of depression. People with no history of mood disorders might experience depressive episodes triggered by stressful events like losing a loved one, the end of a relationship or divorce, and some major life changes like shifting homes or losing a job. The study's design makes it challenging to evaluate a potential link between stressful life experiences and the advent of PPD. Retrospective study design poses the disadvantage of subjects over-reporting an experience, sometimes subconsciously thinking it to be a probable root of illness. However, the bias can be removed by employing a prospective data collection method [49]. A 2005 study by Boyce et al. observed a significant correlation between the risk of PPD and the occurrence of a stressful life event in the past year, with the odds ratio coming out to be 3.14 [26]. Another study by O’Hara et al. found a moderate association between the two; however, based on the place of study, the strength of the association differed greatly. The correlation was strong in the regions of Great Britain and North America, but it was not significant for the Asian regions [36].

Studies have indicated that women who had marital issues while pregnant had a higher chance of PPD. This would manifest emotions of loneliness and a lack of support. Marital relationships and spousal support are among the most essential factors influencing PPD. Spousal support helps lessen the strain on new mothers, thus mitigating the risk of PPD [49]. A study conducted in India found the incidence of PPD to be lower in mothers with greater spousal support and greater in women with unsupportive spouses [17]. Support from partners is found to be protective against the development of PPD. Additionally, there is a continuous correlation between increased risk of PPD and support received from a spouse, satisfaction with said supportive measures, as well as unmet expectations throughout the transition to parenthood. Adequate spousal support throughout the pregnancy also acted as a safeguard against the escalation of pregnancy depression symptoms into postpartum [50-52].

Research done on PPD often takes into account the mother's interpersonal relationships and potential protective elements. Thus, there is strong evidence that the strength and quality of a woman’s perinatal relationships and the network of social support during pregnancy influence the risk of PPD, with healthy, supportive relationships acting as a protective factor, while violence, abuse, conflicts in relationships, and lack of social support increase risk [50]. Familial support is especially of great importance in Asian countries like India. Potential sources of social support include family members, coworkers, and friends. Support may manifest in several ways, such as instrumental, emotional, and informational. Studies have repeatedly demonstrated a decreased risk of PPD in the presence of instrumental and emotional support [49,53]. The meta-analysis by O’Hara et al. concluded that a lack of social support is among the most potent factors influencing PPD [36].

Several studies have provided conclusive evidence of abuse and violence as being risk factors for PPD. Pregnant women suffering from abuse are more likely to suffer from the effects of the said trauma, psychologically rendering them more vulnerable and sensitive to PPD. Often, those suffering from violence may develop a feeling of shame, be influenced by cultural factors, and not seek timely help, leading to negligence of their circumstances. Depressive symptoms could be a response to violence and a covert cry for assistance. These two factors may help explain why violence raises the likelihood of postpartum depression. Further study into the underlying mechanisms is required [20].

Obstetric and pediatric factors

Over the past several years, gestational diabetes has become more common, opening the door to large-scale studies on diseases connected to the condition. Gestational diabetes can be considered a risk factor for PPD, although the impact is small. The exact mechanism by which gestational diabetes results in PPD is still not entirely understood. Pregnancy-related diabetes is a stressful experience that can potentially increase the incidence of postpartum depression by itself. However, insulin resistance and inflammatory responses have also been implicated as potential mechanisms [20,54-56].

Some studies have concluded that despite there being less evidence from current research indicating a link between cesarean delivery and postpartum depression, women who had emergency caesareans were more likely to experience postpartum depression [49]. However, other studies, like the one by Zhao et al., indicate that having a cesarean section, whether an emergency or elective procedure, increases the likelihood of developing postpartum depression, which can exacerbate patient stress. The mother's level of interleukin 6 may rise after a cesarean section, and since interleukin 6 is a key cytokine alteration associated with depression, it is the mediating mechanism between the two [20].

The effects of vitamin D insufficiency in pregnancy and its role in pregnancy-related depression and other negative perinatal effects have only recently been studied. A study by Accortt et al. investigated the association between vitamin D deficiency and the risk of PPD, using vitamin D metabolite ratio (VMR) to measure vitamin D levels, concluding that vitamin D deficiency is a risk factor for PPD [57].

Multiple births and negative birth experiences were also associated with a higher risk of PPD [20]. There have also been studies showing an increased risk of PPD in mothers with preterm and low birth weight infants, especially in the first year postpartum, as compared to those who carried them to term. The probable cause of this association is increased stress over the infant’s health and the risk of diseases or complications [58].

Cultural factors

In Asian nations like India, Vietnam, and China, among others, and in some African nations like Egypt, the birth of a female child has also been identified as a risk factor. There exists a gender preference for male children as they are considered to be the future breadwinners of the family. In most cultures, tradition dictates that the male child takes care of the family economically and supports the parents in their old age. In contrast, the female child is considered a burden, especially in the economically weaker sections, and is regarded as part of the groom’s family post-marriage [19,24].

## Conclusions

Postpartum depression is one of the major health issues that women face. However, a large number of cases go undiagnosed and untreated due to a lack of knowledge and awareness. Social stigma also plays a major role, as women hesitate to get help. The risk factors for postpartum depression are numerous and may have strong to weak associations with the development of PPD. A previous history of depression or psychiatric illness, depressive symptoms during pregnancy, and a low level of spousal and social support were the most powerful factors. Other significant factors include complications during pregnancy, low socioeconomic status, and stressful life events.

According to about 16 studies, the major risk factors were a lack of spousal and social support, previous psychiatric illnesses, gestational diabetes, a negative birth experience, preterm deliveries, low birth weight infants, and a history of abuse. Some studies found that vitamin D status was correlated, while others found no such association. While immigration was found to be a risk factor, it was in turn influenced by factors like social support and socioeconomic position. Lower socioeconomic factors, body image dissatisfaction, pre-pregnancy obesity, cesarean section, and multiple births were also found to have a consistent, albeit weak, correlation with PPD. Studies analyzing maternal age and chronic illness as risk factors were inconclusive, while those studying genetic and epigenetic markers found weak correlations, requiring further investigation. In some countries, the gender of the baby has also been deemed a risk factor due to cultural influences. Although several studies have been conducted, evidence of the strength of association and information regarding the above risk factors is still lacking. Thus, there is a need to conduct more large-scale studies spanning different countries and integrate obtained information to be applicable across all cultures. This would be useful in implementing appropriate preventive measures and providing appropriate therapy to mothers.
